# Too bad! Fixed dose combination antiretroviral drugs

**DOI:** 10.1186/1471-2334-14-S2-P80

**Published:** 2014-05-23

**Authors:** Thomas Agyarko-Poku, Yaw Adu Sarkodie, Linda Atakorah-Yeboah

**Affiliations:** 1 Suntreso Government Hospital, Ghana Health Services, Kumasi, Ghana; 2 Department of Clinical Microbiology, School of Medical Sciences, University of Science and Technology, Kumasi, Ghana; 3 Manhyia Government Hospital, Ghana Health Services, Kumasi, Ghana

## Introduction

Pill burden is a factor for non-adherence to Antiretroviral drugs (ARVs). The introduction of Fixed Dose Combination ARVs regime is seen as an antidote to pill burden among HIV patients on treatment. The study hypothesized that not all HIV patients will accept this new regime, and was conducted to determine their perception on the new treatment regime.

## Materials and methods

1681 HIV positive patients accessing care at Suntreso STI/HIV Clinic in Kumasi, who have been on treatment for more than 12 months and consented to participate were recruited for this cross sectional study. They were interviewed using semi-structured pre-texted questionnaire prior to the commencement of the new fixed dose combination ARVs treatment regime. Data was entered and analyzed using SPSS version 16.

## Results

Whereas, 60.9% (1023/1681) find the present dose regime cumbersome, 39.1% (657/1681) of the patients prefer the multiple dose regime. 42.2% (709) of respondents have some reservations about fixed dose combination regime whilst 24.6% (413) prefer it, with 33.3 % (559) being in different. Reasons for the reservation included; ‘Side effect may be too serious’ (48.3%, 342/709), ‘Virus too powerful for a single molecule’ (30.2%, 214/709), ‘Attempt to deprive us of drugs and facilitate our death’ (13.8%, 92/709) and ‘Cost of drugs will be expensive in future for a combine drugs therapy’ (7.7%, 55/709).

## Conclusions

Although, majority of patients find the multiple dose regimes cumbersome, they are skeptical about the use of the fixed dose combination treatment regime. The new regime may result in overdosing if they find it inadequate to provide the needed protection. The fear of serious adverse reaction from combination of ARVs compared with separate drugs may scare them from taking the treatment. Intensive adherence counseling taking care of the above concerns is essential before patients are switched onto the fixed dose ARV regime.

